# Functional genomics of simian malaria parasites and host–parasite interactions

**DOI:** 10.1093/bfgp/elz013

**Published:** 2019-06-26

**Authors:** Mary R Galinski

**Affiliations:** 1 Emory Vaccine Center, Yerkes National Primate Research Center, Emory University, Atlanta, GA, USA; 2 Division of Infectious Diseases, Department of Medicine, Emory University School of Medicine, Atlanta, GA, USA

**Keywords:** Malaria, *Plasmodium knowlesi*, *Plasmodium cynomolgi*, *Plasmodium coatneyi*, nonhuman primates, functional genomics, systems biology

## Abstract

Two simian malaria parasite species, *Plasmodium knowlesi* and *Plasmodium cynomolgi*, cause zoonotic infections in Southeast Asia, and they have therefore gained recognition among scientists and public health officials. Notwithstanding, these species and others including *Plasmodium coatneyi* have served for decades as sources of knowledge on the biology, genetics and evolution of *Plasmodium*, and the diverse ramifications and outcomes of malaria in their monkey hosts. Experimental analysis of these species can help to fill gaps in knowledge beyond what may be possible studying the human malaria parasites or rodent parasite species. The genome sequences for these simian malaria parasite species were reported during the last decade, and functional genomics research has since been pursued. Here research on the functional genomics analysis involving these species is summarized and their importance is stressed, particularly for understanding host–parasite interactions, and potentially testing novel interventions. Importantly, while *Plasmodium falciparum* and *Plasmodium vivax* can be studied in small New World monkeys, the simian malaria parasites can be studied more effectively in the larger Old World monkey macaque hosts, which are more closely related to humans. In addition to *ex vivo* analyses, experimental scenarios can include passage through Anopheline mosquito hosts and longitudinal infections in monkeys to study acute and chronic infections, as well as relapses, all in the context of the *in vivo* host environment. Such experiments provide opportunities for understanding functional genomic elements that govern host–parasite interactions, immunity and pathogenesis in-depth, addressing hypotheses not possible from *in vitro* cultures or cross-sectional clinical studies with humans.

## Introduction

Human infections with simian malaria parasites have been confirmed in Southeast Asia, given the availability and strategic use of genomic technologies [1–3]. While their numbers remain relatively small compared to the hundreds of millions of human cases of malaria caused annually by *Plasmodium falciparum*, *Plasmodium vivax*, *Plasmodium ovale* and *Plasmodium malariae* [4], the zoonotic cases can cause illness and morbidity, there have been cases of death caused by *Plasmodium knowlesi* malaria [5–8], and the possibility of severe illness caused by *Plasmodium cynomolgi*. *Plasmodium cynomolgi* can cause a range of illness manifestations, from mild to severe, in *Macaca mulatta* (of Indian origin) [9, 10], a host that can be infected with this species in nature [11, 12]. Therefore, as with *P. knowlesi*, the chance for severe *P. cynomolgi* malaria in humans cannot be discounted, and this species must stay on the clinical radar when one is diagnosing and treating patients in areas of the world where *P. cynomolgi* has been detected.

*Plasmodium knowlesi* was identified as a zoonotic species of public health importance in 2004 in Malaysia based on using polymerase chain reaction (PCR) amplification methods and gene sequence analysis, and it has since been noted as the main cause of malaria in areas of Malaysia and a threat in many neighboring countries [1, 7, 13, 14]. This species had previously been mistaken for *P. malariae,* due to similarities in appearance by light microscopy. *Plasmodium knowlesi* is transmitted in the region by the *Anopheles* leucosphyrus vector group to Old World monkey hosts, specifically *Macaca fascicularis* (also known as long-tailed macaques, crab-eating or kra monkeys) and *Macaca nemestrina* (also known as pig-tailed macaques), and subsequently to humans particularly living or working near the forest fringe [15, 16].

Until this year, there was only one case of zoonotic *P. cynomolgi* malaria confirmed, in Malaysia [17], but recent active surveillance of malaria in individuals using PCR methods has led to the confirmation of 5 cases of *P. cynomolgi* malaria in hospitals and clinics around Kapit [18] in Sarawak, Malaysian Borneo, and in 23 villages in Pailin and Battambangin western Cambodia has resulted in the confirmation of 13 asymptomatic cases with *P. cynomolgi* [3]. These instances of *P. cynomolgi* zoonotic cases may be an indicator of the wider presence of this zoonosis, perhaps overlooked as *P. vivax* in the past, based on very similar morphology viewed by microscopy. This has important epidemiological implications with regards to a dormant parasite reservoir and relapses, as discussed below, and lends support to the use of *P. cynomolgi* as a model to study *P. vivax*. *Plasmodium cynomolgi,* like *P. knowlesi*, is transmitted by the *Anopheles* leucosphyrus vector group, and others [11, 12], to *M. fascicularis* and *M. nemestrina*. For both parasite species, there have so far been no confirmations of infections cycling solely between mosquitoes and humans*.* Several other simian malaria parasite species exist in the area (e.g. *Plasmodium coatneyi*, *Plasmodium fragile*, *Plasmodium fieldi* and *Plasmodium inui*) [19–21], but these have yet to be found in humans.

Here, an overview is provided of functional genomics studies accomplished with *P. knowlesi* and *P. cynomolgi*, and their importance, with reference to the possibilities and benefits for such continued research. *Plasmodium coatneyi* is also highlighted. These and other simian malaria parasite species have been informative as model parasites and complement research on *P. falciparum* and *P. vivax,* which cause the vast majority of illness in humans [4]. Basic biological knowledge about each of these species can be found in the foundational book titled ‘The Primate Malarias’ [11, 12]. The phylogenetic relationships have been reviewed recently by experts in evolutionary biology [20, 21], and key details about established *Plasmodium* species–nonhuman primate (NHP) host experimental model systems have been summarized elsewhere, including in a book titled ‘Nonhuman Primates in Biomedical Research’ [22]. Studies utilizing simian malaria parasites over many decades have been instrumental to malaria research and in many cases groundbreaking (e.g. [23–28], and reviewed in [29–32]). The future is wide open for the expanded use of these parasite species and NHP infections in malaria functional genomics and systems biology research [33–36]. One may ask, why bother—versus analyzing human clinical samples and rodent models? And, it has been said that NHP studies are expensive. Moreover, only a few labs can work with these models, given the requirement for specialized expertise and resources. Yet collaborations have and can form to make these models more accessible. And, there is no doubt that simian malaria parasites and NHP model systems have contributed to malaria research immensely over decades and facilitated scientific breakthroughs as well as the development and testing of interventions. So, why bother? Why not?

### Functional genomics of NHP malaria parasites and model systems—where to begin

Comparative genomics and functional genomics research involving primate malaria parasite species has been advancing on the two most predominant human malaria parasite species, *P. falciparum* and *P. vivax* (reviewed in [33, 37], and articles in this special issue)*,* and simian malaria parasite species, including *P. knowlesi*, *P. cynomolgi* and *P. coatneyi,* elaborated in this article. Isolates of each of these simian species (and strains when available) were stored and propagated in monkeys for research at the US Centers for Disease Control and Prevention [38] and these have been distributed during the past 20 years by the Malaria Research and Reference Repository (MR4), which was established by the US National Institutes of Allergy and Infectious Diseases and is currently administered by BEI Resources (managed by the American Type Culture Collection, ATCC) [39]. Strains that are most commonly used in current research are also typically stored as cryopreserved isolates in various laboratories, noted in publications. For this research to continue now and over time, it will be important for such stocks to be maintained, characterized and made available as needed to future research groups. The importance of preserving these stocks and any future monkey, human or culture-adapted collections, along with their life histories and quality control checks (e.g. sequencing confirmations), cannot be over stressed. It would indeed be useful for the ATCC, or others, to commit to such tasks for the foreseeable future, especially now while functional genomics and systems biology technologies are enabling in-depth discovery that has been unprecedented and can be groundbreaking. A demand with stepped up inquiries for such resources may make this possible. Moreover, collaborative arrangements are strongly encouraged to maximize the use of these species and strains, pairing malaria experts with the necessary resources, knowledge and experience, with others who can bring added value from various scientific disciplines including functional genomics.

Comprehensive annotated genome sequences were reported within the past 10 years for *P. knowlesi* [40, 41] (Malayan strain; this was mistakenly reported and propagated for decades in the literature as the H strain [42]) and *P. cynomolgi* [43, 44] (B/M strain; also referred to in the literature as the NIH, B, Bastianelli, M, or Mulligan strain) and recently in 2016 the 1st genome sequence was reported for the simian malaria parasite species *P. coatneyi* [45] (Hackeri strain; this is the only strain preserved for this species) [11, 12]. By contrast, and to put this conversation into perspective, the 1st genome sequence for *P. falciparum* (3D7 strain) was reported in 2002 [46]. Functional genomics studies for this species are naturally most advanced [33] (reviewed in [33, 47–51]), and this progress is discussed in several articles in this ‘Briefings in Functional Genomics’ special issue. Functional genomic studies on *P. falciparum* have had the benefits of robust *in vitro* blood-stage culture capabilities and access to *P. falciparum* clinical samples from many malaria endemic areas worldwide. Functional genomics research has also been developing for *P. vivax* since the Salvador I strain genome sequence was reported in 2008 [52], and involving some patient samples [53–57], but without the benefit of robust blood-stage culture systems [58, 59]. A summary of the state of genomics for each of these human malaria parasite species and others, including new isolates and strains, has been reported recently by Garrido-Cardenas and colleagues [37].

Individual gene and genome sequences have been studied to show phylogenetic relationships of the primate malaria species and strains (e.g. see [60–62]), and now, functional genomics analyses are beginning to show the additional benefits of looking beyond the parasite genome sequences when seeking targets of interventions, and specifically, the benefits of comparative analysis across species. For example, the functional expression of the parasite’s genome throughout its complex life cycle in vertebrate and invertebrate hosts can reflect species-specific adaptations as well as essential conserved functions that may point to new targets for interventions [63]. Understanding epigenetic regulation and gene expression throughout the parasite’s life cycle is important, including distinguishing mechanistic steps and controls at the nuclear and cytoplasmic levels [64–66]. Ultimately, there is much to be learned about the gene expression and regulation similarities and differences for each of these species and throughout their life cycles, including transcriptional and post-transcriptional regulation, the roles of epigenetics and non-coding (nc) RNAs and possible controls at the level of mRNA translation and protein expression. Relatively speaking, this research is in its infancy, yet it is beginning to advance with the increasing availability and use of sophisticated enabling technologies.

Critically, host factors have been implicated in the maintenance of certain parasite biological characteristics *in vivo*, e.g. relating to virulence, and specifically parasite gene expression regulatory mechanisms [67–69]. From this standpoint, though often overlooked in favor of the evolutionarily more distant mouse models (due to their comparatively ease of use and feasibility by many, as well as utility; reviewed in [33, 70, 71]), the simian malaria parasites remain highly relevant. These parasites and their host are more closely related to the human malaria parasite species and human hosts, respectively. One can study them using *in vivo*, *ex vivo* and *in vitro* NHP infection models and systems, with direct relevance for improved understanding of the human parasite species and preventing or treating disease (reviewed in [29, 70–72]). Over the past 6 years, longitudinal NHP infection experiments were performed by the Malaria Host-Pathogen Interaction Center (MaHPIC) to dynamically study and model parasite–host interactions using systems biology approaches and release large inter-related datasets for further analysis by the research community. These efforts are summarized in the PlasmoDB reference database [73] including experiments with *P. cynomolgi* or *P. coatneyi* in *M. mulatta* [10, 74–76], *P. knowlesi* in both *M. mulatta* and *M. fascicularis*, and *P. vivax* in *Aotus nancymae* and *Saimiri boliviensis* monkeys [77–79]. Genome sequences are available for each of these host species [80–84], and numerous datasets and metadata are now publicly available from the MaHPIC experiments at the PlasmoDB website for further analysis by the research community. Included are in-depth host transcriptomes and parasite transcriptome datasets, as well as proteomic, lipidomic, metabolomic, immune profiles, clinical and parasitological data to allow functional genomics and systems biology analysis in the course of longitudinal infections. These detailed NHP experimental infections were initiated with sporozoites, thus allowing analysis of infections from the liver stage through the blood stage and different degrees of illness, with the capture of possible changes in gene expression due to the presence of physiological changes including host immune responses. Future studies could include functional genomic analyses from NHP to the vector host and back to NHP, to gain knowledge of the host–parasite gene expression and epigenetic regulation throughout the *in vivo* life cycle and infection periods.

*Plasmodium vivax* and *P. falciparum* can be studied in the small New World monkey hosts (e.g. see [79, 85–87]), albeit with limitations compared to macaque infections, due to the small size of New World monkeys, lower and resolving parasitemias, blood volume limitations, and the inability to acquire multiple bone marrow samples. Moreover, while some do, most strains that have been adapted to the blood of these animals will not productively infect mosquitoes and yield sporozoites (reviewed in [88]); similarly, most strains of *P. falciparum* adapted to long-term *in vitro* culture do not produce gametocytes that will productively infect mosquitoes, and there is no comparable *in vitro* culture system yet in place for *P. vivax* [89]. Nevertheless, these species are very important animal models for specific experimental questions and for validation of findings obtained from macaque infections with the most apropos species [11, 12]; e.g. *P. coatneyi* to mirror *P. falciparum*; *P. cynomolgi* to mirror *P. vivax*; and *P. knowlesi* for specific questions, for example, relating to mechanisms of antigenic variation (reviewed in [72]).

For all primate malaria species (whether human or simian), functional genomics studies on *Anopheles* vectors, insect stages and host-parasite interactions hold much value (e.g. see [90–93]), and likewise for liver-stage parasites, and these are beginning to advance with new technologies including for liver-stage cultures (in particular, see below, relating to *P. cynomolgi* and *P. vivax* advances on hypnozoite research), and acquisition of purified samples and RNA analysis from relatively few or single cells [94, 95].

### Plasmodium knowlesi

*Plasmodium knowlesi* has proven for decades to be an excellent experimental model parasite (reviewed in [29, 30, 72]) and, as noted above, the Malayan strain genome sequence has been available [40, 41]. Advanced sequencing technologies are now permitting the development of whole-genome sequence data from *P*. *knowlesi* isolates [42, 96]. One study based on 48 clinical infections and 5 laboratory-maintained lines highlights this species’ high degree of genetic diversity and population substructure clusters [42]. Among the simian malaria parasite species, *P. knowlesi* is most closely related to *P. coatneyi* [97]*,* which as discussed below shares asexual stage morphological and biological features with *P. falciparum,* including a 48 h life cycle, infected red blood cell (RBC) knobby protrusions, cytoadhesion and deep vascular sequestration characteristics [11, 12]. Curiously, on the other hand, *P. knowlesi* has a 24 h asexual blood-stage life cycle *in vivo*, and while the infected RBCs have some adhesive capabilities [98, 99], they do not cytoadhere and sequester like *P. falciparum*. Regardless, *P. knowlesi* is known to undergo antigenic variation of specific large proteins that are expressed at the surface of the infected RBCs, comparable to *P. falciparum* (discussed below, and reviewed in [72]).

*Plasmodium knowlesi* can be grown robustly in *in vitro* cultures within rhesus RBCs and it is amenable to genetic manipulation *in vitro* as well as *in vivo/ex vivo* [100, 101]. Recently *P. knowlesi* was adapted to grow *in vitro* in human RBCs and used effectively in these host cells for transfection studies [102–105], and the genome from these adapted parasites was sequenced with over 40 000 predicted methylation sites defined [106]. The methylome provides another layer of complexity to the *P. knowlesi* genome and predicted epigenetic regulation of gene expression*,* as first reported for *P. falciparum* [107]. Other recent work included *P. knowlesi* data in a comparison of heterochromatin protein 1 (HP1) occupancy across primate and rodent malaria parasite species and demonstrated that HP1-dependent gene silencing is evolutionarily conserved in malaria parasites [108]. These findings are highly relevant with regards to understanding gene expression control in *Plasmodium* and parasitic mechanisms of antigenic variation, invasion of host cells and conversion to gametocytes. Progress is being made in each of these areas, yet we are still in the early stages of grasping the complexity of each mechanism and the regulatory role of host factors in the course of infections.

Recent biological analyses of *P. knowlesi* grown *in vitro* in human RBCs include a comprehensive stage-specific multi-modal microscopic assessment of the parasite’s organelles, internal membraneous and surface structures, while emphasizing the importance of this knowledge with regards to pathology in human cases. Several comparisons were also made by this team with the biology of *P. falciparum* or *P. vivax* infected RBCs [109]. One can draw from such studies elements that are shared and unique across the species and gain new perspective on the value of *P. knowlesi* as both a human pathogen and a model parasite. A related study has also been published on *P. knowlesi*-infected rhesus monkey RBCs [110]. Together, these studies provide an advanced biological view of *P. knowlesi* blood-stage forms, insightful comparisons across species, and a new framework for applying knowledge gained from functional genomics.

Several studies by Lapp and colleagues have focused on functional genomics of *P. knowlesi* in relation to understanding the molecular, immunobiological and genetic mechanism that govern the schizont-infected cell agglutination (SICA) variant antigen (*SICAvar*) expression *in vivo*, building upon the groundbreaking studies by Brown and Brown reported in 1965 [23] that showed using the rhesus macaque infection model that antigenic variation occurred in blood-stage infections (reviewed in [72]). In particular, RNA and protein analyses showed that two *P. knowlesi* clones (Pk1(A+) and Pk1(B+)1+), derived one from the other *in vivo* as a result of antigenic variation [111], had completely different repertoires of *SICAvar* gene and SICA protein expression [69]. Additionally, in SICA[−] clones derived from passage in splenectomized monkeys and shown to lose virulence [68], *SICAvar* expression was downregulated and no SICA protein was identified [69]. Other work examining the 24 h intra-developmental cycle transcriptome using microarrays demonstrated that there were major differences in gene expression in *in vitro* compared to *ex vivo* time course samples [112]. This study showed dramatic downregulation of the *SICAvar* gene family in the *in vitro* samples, similar to observations from SICA[−] infected RBCs generated in splenectomized hosts [69]. On the other hand, genes encoding proteins considered important for merozoite invasion of RBCs had comparable expression [112]; this bodes well for continued *in vitro* investigations on mechanisms of merozoite invasion of host cells. Hoo and colleagues continued to assess these data in a multi-species gene expression comparison study and determined that the expression of certain sets of genes are more or less conserved at different stages of the asexual life cycle [113].

Recently, to identify conserved and species-specific principles of genome organization, Bunnik and colleagues generated 3D genome models via Hi-C analysis for five species of *Plasmodium* including *P. knowlesi* [63]. In *P. knowlesi*, telomeres and virulence genes were more dispersed throughout the nucleus, compared to the other species (*P. falciparum*, *P. vivax* and two rodent parasite species). Still, its 3D genome architecture likewise showed a strong correlation with gene expression. In particular, these data show that nuclear genome organization is constrained and that the *SICAvar/var* virulence genes in *P. knowlesi* and *P. falciparum*, respectively, have an effect on chromosome folding [63].

Going forward, directional RNA-seq has the potential to provide a more detailed view of gene expression across the *P. knowlesi* intraerythrocytic development cycle, and to confirm the presence of specific sense and antisense nc RNAs, and importantly whether they are associated with the ON or OFF states of *SICAvar* genes (reviewed in [72]), and if they have functional roles similar to those being unraveled in functional studies of *P. falciparum var* gene expression [92, 114, 115]. Furthermore, the time is ripe for functional genomics analysis of *P. knowlesi* in the context of the rhesus monkey where extreme virulence is the norm, and its natural human and NHP hosts where resilience is the norm (though severe situations are possible, as indicated above with regards to zoonotic infections) (reviewed in [29, 30]). Longitudinal systems biology infection experiments can begin to discern the physiological and immunological host factors and cascades that result in one outcome or another.

### Plasmodium cynomolgi

*Plasmodium cynomolgi* is closely related to *P. vivax* [43, 44, 60, 61] and there is growing interest in utilization of this species as a model to identify targets of intervention against *P. vivax.* Notably, the asexual blood stages of both species develop in approximately 48 h and with caveolae vesicle complexes in abundance around the entire RBC membrane [116, 117]; these differ greatly from the surface structures of other species featured in this review (e.g. see [109, 110]). Life cycle time course studies with omics data have yet to be reported for *P. cynomolgi*, though these and comparisons across species would be of extreme value for better understanding the distinctive biology of each and possible conserved and unique targets of intervention.

An initial *P. cynomolgi*-rhesus monkey longitudinal infection transcriptome study was based on microarray technology with the analysis of whole-blood gene expression during both liver- and blood-stage periods [118]. Interestingly, this sporozoite-initiated infection experiment noted distinctive differences in the host response during each phase of the infections. Recent longitudinal infections in rhesus macaques, also initiated with sporozoites and showing primary and relapse infection outcomes [10], have been analyzed using multi-omic data sets to distinguish bone marrow changes in response to acute primary and relapsing infections and features of mild and severe disease, dynamically model metabolic pathways, and develop new methods for the analysis and integration of multi-omic datasets. Specifically, Tang, Joyner and others studied longitudinal clinical and immune profiles along with bone marrow transcriptomic data and determined that malarial anemia may be driven by monocyte-associated disruption of GATA1/GATA2 function in erythroid progenitors during acute but not relapse infections [74]. Yin and colleagues used transcriptomic and immune profiling data from the same *P. cynomolgi* cohort infections to distinguish disease severity [119]. Also based on the same infection cohort, Tang and colleagues showed flux redistributions within the purine pathway that were also consistent with data from *P. coatneyi* infection of rhesus macaques and human *P. falciparum* infections [120]. Most recently, transcriptomic, metabolomic and lipidomic data from this cohort were used to analyze relationships across several biological layers utilizing a mutual information-based machine learning approach to integrate heterogeneous longitudinal datasets and construct an atlas of multi-omics relatedness networks [121]. Future longitudinal systems biology studies with multiple NHP host species would be of interest, e.g. with *M. fascicularis* and *M. fuscata*, where *P. cynomolgi* infections have been shown to have different clinical spectrums [122]. Additional directions could include co-infection and drug testing studies, e.g. as performed previously with *P. cynomolgi* and SIV in rhesus monkeys [123, 124].

Breakthroughs in culturing *P. cynomolgi* blood-stage parasites are on the horizon [160] and these have provided glimmers of hope in relation to expansion of use of this model species, particularly to advance research on liver-stage biology and the identification of drug targets and novel interventions. The Bill and Melinda Gates Foundation has taken notice and recently supported a meeting of experts who are advancing this line of research [125]. *Plasmodium cynomolgi* blood-stage cultures hold potential for the future infection of mosquitoes from cultures rather than infected blood from NHPs. If this is achieved, with the normal production of all insect stages of the parasite including infectious sporozoites and then liver-stage forms in culture, functional genomics investigations based on the full life cycle of this species will be more accessible, and together with the strategic use of *in vivo* models, the prospects will be strengthened for identifying novel liver-stage targets for future interventions against *P. vivax.* Both species, *P. cynomolgi* and *P. vivax*, harbor dormant parasite forms in the liver called hypnozoites [126–129] that can become activated weeks or months after a primary blood-stage infection and cause relapsing episodes of malaria. This dormant reservoir and relapses with coincident ongoing transmission possible are major impediments to the regional elimination and ultimate eradication of malaria worldwide (reviewed in [31, 130–134]).

In the past 2 years, multiple research teams have developed preliminary transcriptomes of cultured *P. cynomolgi* and *P. vivax* liver-stage parasites, based on each of their unique liver culture systems [55, 135–137]. Cubi and colleagues first reported hypnozoites transcriptome data based on laser capture microdissection isolation of *P. cynomolgi* infected *M. fascicularis* hepatocytes (hypnozoites and schizonts); they compared the transcriptomes of the dividing and quiescent parasites and honed in on specific differentially expressed transcripts and molecular pathways that may in part distinguish the biology of hypnozoites and their activation [135]. Voorberg-van der Wel and colleagues then put forth a comparative analysis of replicating and dormant *P. cynomolgi* parasites [136]. They distinguished a smaller set of differentially expressed transcripts attributed to hypnozoites and delved into pathways that may lead to new drug targets. Recently Bertschi and colleagues from this team [137] went a step further and revealed a 10-fold decrease in gene expression when *P. cynomolgi* liver-stage parasites matured into the dormant stage. Transcription from a limited set of 840 genes identified from purified hypnozoites showed the maintenance of basic metabolic housekeeping functions, and pathways pertinent to genome integrity, energy metabolism and quiescence [137]. Working directly with *P. vivax* infections, Gural and colleagues [55] developed initial transcriptomes from cultured liver-stage forms, notably obtaining a reduced transcriptional profile from hypnozoite-enriched samples compared to samples with mixed stages; a subsequent paper by Roth and colleagues [56] is also worth noting, as it reports the development and testing of methods for cultivating the *P. vivax* liver-stage forms in a 384-well system for drug testing and these may also prove useful for future stage-specific liver-stage parasite isolations and omic analyses. Together these papers are showing progress toward understanding the basic nature of the hypnozoite, and the biology of dormancy and activation of these forms. Early studies performed to establish *P. cynomolgi* liver-stage culture systems capable of developing all of the active and quiescent life stages suggested epigenetic regulation plays a role [28, 138]. With continued advancement of technologies and proof-of-principle studies showing feasibility, researchers are becoming poised to determine the biochemical and epigenetic regulatory mechanisms and processes that enable the successful short- and long-term parasitism of *P. cynomolgi* liver-stage parasites, and by inference if not direct investigation, *P. vivax* in the liver. This area of research has been a mysterious and largely overlooked ‘black box’ since the discovery of hypnozoites over 35 years ago [126–129], but global eradication goals from the past 10 years have spurred on a scientific race to reveal the mysteries of dormancy, and as a result the prospects for eventual new drug targets and interventions targeting hypnozoites have changed from being gloomy to propitious.

### Plasmodium coatneyi

The asexual RBC stages of *P. coatneyi* have morphological and cyclical growth characteristics similar to *P. falciparum*, including a 48 h life cycle, infected RBC knobby protrusions, cytoadhesion and deep vascular sequestration [11, 12, 139]. This species has therefore been studied in multiple species of macaques as a model for *P. falciparum* malaria, pathogenesis and anemia in naïve and immune individuals [75, 140–147], and also to study cerebral malaria [139, 148–150] and malaria during pregnancy [151–154] (reviewed in [155]). Other *P. coatneyi* infection studies have involved comparative pharmacokinetics and pharmacodynamics drug testing in rhesus monkeys [156], the dynamics and immune responses of *P. coatneyi* and *Shistosoma mansoni* co-infections in rhesus monkeys [157] and assessment of *P. coatneyi* liver-stage parasites in *S. boliviensis* monkeys [158]. Functional genomic studies are just beginning with this parasite species. Recent longitudinal infection experiments with *P. coatneyi* in a cohort of rhesus macaques with a focus on high-resolution metabolomics has shown the value of functional genomics in the assessment of host–parasite interactions during the course of infections and specifically demonstrated the differential expression of *Plasmodium* genes as infections became chronic [159]. Based on this same cohort, as noted above, flux redistributions within the purine pathway were shown to be consistent with data from *P. cynomolgi* infection of rhesus macaques and human *P. falciparum* infections [120].

As noted above, there is currently only one strain of *P. coatneyi* available (the Hackeri strain) and its genome sequence was published in 2016 [45]. This was the first reported *Plasmodium* genome sequence based on Pacific Biosciences Technologies, allowing long-read sequences. This is important since like the closely related species *P. knowlesi, P. coatneyi* has a large complex multi-exon variant antigen *SICAvar* gene family, with anticipated comparable gene and protein regulatory processes that govern antigenic variation. While currently expression of the *SICAvar* gene family is most amenable to study with *P. knowlesi* infections of macaques (reviewed in [29, 72]), it could be studied as well using the *P. coatneyi*–macaque infection model, along with the integration of clinical and pathology data related to this species’ deep vascular sequestration. *Plasmodium knowlesi* has been the preferred species for in-depth studies of antigenic variation since parasite clones and *in vivo SICAvar*/SICA switched phenotypes were generated for this species (reviewed in [29, 72]). Going forward, generating comparable clones and studies with *P. coatneyi* may prove to be useful; regardless, there is great potential for functional genomics advances with *P. coatneyi*, building upon extensive knowledge gained from the variety of *in vivo* NHP infection experiments detailed above.

### Concluding remarks

This review has focused on simian malaria parasite species, emphasizing how they can be informative as model parasites and complement research on other species, especially *P. falciparum* and *P. vivax.* Aside from the wealth of knowledge that can be gained from individual species and comparative studies, knowledge of each species can become important in the context of testing future possible interventions in NHP hosts. Moreover, in-depth multi-omic and clinical, immunological and pathology data are possible from longitudinal studies and together allow for systems biology analyses with the benefit of machine learning and mathematical modeling approaches [33–36] that may yield predicted new targets and solutions for novel interventions. Functional genomics and systems biology studies may also yield interesting insights into circadian rhythms and their importance relative to host–parasite interactions and responses, and the conundrum of how *P. knowlesi* completes its blood-stage cycle in about 24 h compared to about 48 h for each of the other species discussed in this review. Such knowledge may help point to conserved mechanisms across the species that could be considered as new targets of intervention against the parasite, or to modulate the host responses. Hypothesis-driven questions are arising from various published studies that can be delved into with experimental designs that allow increasing depth into areas of interest, for example to understand and possibly manipulate the host’s physiological response to infection (or drugs), the immunological functioning of different organs and cell types to control the disease, and the transmissibility of infections.

#### Key Points

- *Plasmodium knowlesi* and *P. cynomolgi* are simian malaria parasite species and causative agents of zoonotic malaria in Southeast Asia, which also serve as model organisms for the main human malaria parasite species *P. falciparum* and *P. vivax,* respectively*.*
- Comparative functional genomics involving these and other diverse simian malaria parasite species such as *P. coatneyi* (a model for *P. falciparum*) across their life cycles can provide novel insights on species-specific and cross-species essential biology and possible points of intervention.
- Critically, these species can be studied effectively in longitudinal infections of NHPs to understand functional genomic elements that govern host–parasite interactions, epigenetic changes, immunity and pathogenesis attributed to malaria.
- Experimental insights can further come from investigating functional genomics of the simian malaria parasites in human red blood cells, in their *Anopheles* sp. vector hosts, from sporozoite inoculation of liver cultures and in multiple NHP host species known to have infection outcomes with different degrees of severity.

## Notes

**Professor Galinski** has over 30 years of experience in malaria research with emphasis on species that cause malaria in humans and nonhuman primates and a recent focus on systems biology as leader of the Malaria Host-Pathogen Interaction Center (MaHPIC).
